# Supplementary material for: An avian femur from the Late Cretaceous of Vega Island, Antarctic Peninsula: removing the record of cursorial landbirds from the Mesozoic of Antarctica
Source: PeerJ. 2019 Jul 10;7:e7231. doi: 10.7717/peerj.7231 (PMC6626523; doi:10.7717/peerj.7231)
Supplement: Supplemental Information 1 — Character matrix for selected taxa, showing only those characters for which scorings could be made for SDSM 78247. [file peerj-07-7231-s001.docx]

Supplemental File for West et al., An avian femur from the Late Cretaceous of Vega Island, Antarctic Peninsula: removing the record of cursorial landbirds from the Mesozoic of Antarctica.

Character states scored for *Vegavis* sp. (SDSM 78247). Characters numbered as in Worthy et al. (2017).

|  | 188 | 193 | 194 | 195 | 198 | 199 | 200 | 201 | 202 | 204 | 205 | 206 | 207 | 209 | 211 | 213 | 214 | 215 | 218 | 219 | 220 |
| --- | --- | --- | --- | --- | --- | --- | --- | --- | --- | --- | --- | --- | --- | --- | --- | --- | --- | --- | --- | --- | --- |
| *Polarornis^1^* | 0 | 0 | 1 | 0 | 0 | 1 | 2 | 0 | 0 | 1 | 0 | 0 | 4 | 1 | 0 | 0 | 0 | 0 | 2 | 0 | 0 |
| *Vegavis^1^* | 0 | 0 | 1 | 0 | 0 | 1 | 2 | 0 | 0 | 1 | 0 | 0 | 4 | 1 | 0 | 0 | 0 | 0 | 2 | 0 | 0 |
| *Vegavis* *iaai^2^* | 0 | 0 | 0 | 0 | 0 | 1 | 2 | 0 | 0 | 1 | 0 | 0 | 4 | 1 | 0 | 0 | 0 | 0 | 2 | 0 | 0 |
| SDSM 78247 | 0 | 0 | 1 | 0 | 0 | 1 | 2 | 0 | 0 | 1 | 1 | 0 | 4 | 0 | 0 | 0 | 0 | 0 | 2 | 1 | 1 |
| *Cariama* *cristata* | 0 | 0 | 0 | 1 | 1 | 1 | 0 | 0 | 0 | 1 | 1 | 1 | 0 | 0 | 1 | 0 | 0 | 1 | 0 | 0 | 0 |

1: from Agnolín et al., 2017

2: from Worthy et al., 2017

Character Descriptions:

188. Femur, proximal end, facies articularis antitrochanterica, lateromedial plane:

0: surface concave (e.g. *Anseranas*);

1: surface convex.

193. Femur, caudal facies, pneumatic openings adjacent to facies articularis antitrochanterica:

0: absent or small;

1: present and large

194. Femur, proximal end, caudolateral margin:

0: impressiones obturatoriae on a large bulbous area close to the facies articularis antitrochanterica extending from the lateral onto the caudal facies and the caudolateral margin further distally lacks further prominences;

1: an elevated impressiones obturatoriae close to the facies articularis antitrochanterica and a large prominence further distally on the caudolateral margin for m. ischiofemoralis that is separated from the former by a sulcus as broad as the impression. , 195

195. Femur, proximomedial part caudal facies, scar for insertion of M. puboischiofemoralis pars medialis:

0: either absent or a weak crest;

1: a strongly developed, elevated, rugose crest, round or elongate.

198. Femur, proximal end, cranial facies, impression of m. iliofemoralis internus:

0: not or weakly marked;

1: well-marked rugosity.

199. Femur, corpus femoris in caudal view, relative length, lateral and medial margins and least width:

0: shaft short, least width at or proximal of mid length, margins not parallel;

1: elongate, least width at mid length, margins parallel for about middle third of length.

200. Femur, dorsoventral curvature of shaft, lateral view:

0: straight or slight;

1: moderate curvature of distal third;

2: strong curvature.

201. Femur, profile of medial facies in caudal aspect:

0: relatively straight or slight curvature;

1: markedly concave.

202. Femur, proximal end, lateral facies, insertion area of m. iliotrochantericus caudalis:

0: located at mid depth;

1: in dorsal half of depth, but separated from dorsal margin;

2: on dorsal margin.

204. Femur, caudal facies, distal half, location of tuberosity or most prominant part crista medialis:

0: towards middle of shaft;

1: on medial margin of shaft e.g. *Genyornis*;

2: on lateral margin of shaft, e.g. *Paracathartes howardae*.

205. Femur, distal end, cranial aspect, orientation of condylus lateralis:

0: little or not divergent from axis;

1: markedly divergent, e.g. dromornithids.

206. Femur, proximal end, lateral facies, area for the insertion of the m. obturatorius medialis proximally and insertion area of the m. ischiofemoralis more distally:

0: widely separated with gap wider than length of insertion of the m. obturatorius medialis;

1: adjacent or narrowly separated with gap less than length of insertion of the m. obturatorius medialis;

207. Femur, distal end, tuber. m. gastrocnemialis lateralis, form:

0: rounded scar, close to or abutting trochlea fibularis on caudal facies;

1: round to oval scar, well separated from trochlea fibularis on caudal facies;

2: an elongate scar/ridge with distinct medial bend, or oriented medially, on caudal facies, may extend proximad of patella sulcus, e.g. most anatids;

3: elongate rugose scar in deep or shallow fossa traversing caudal facies from lateral edge of trochlea fibularis to proximal end of crista tibiofibularis, may be merged with ansa m. iliofibularis caudalis;

4: a rugose scar or shallow fossa on the lateral facies proximocranial to the cond. fibularis.

209. Femur, caudal aspect, trochlea fibularis with distinct depression that is not insertion for m. gastrocnemialis lateralis immediately proximal of the articular surface:

0: not so;

1: yes.

211. Femur, cond. medialis, proportion of maximum distal width:

0: approximately half;

1: clearly greater than half.

213. Femur, distal end, width sulcus patellaris in cranial view, taken at half the depth of the bounding condyles:

0: broad and flat, wider than condylus lateralis;

1: narrow and deep, less than width condylus lateralis plus trochlea fibularis.

214. Femur, the notch, or fovea tendineus m. tibialis, on the distal end of cond. lateralis, in distal view:

0: notch present;

1: notch absent.

215. Femur, distal end, fossa poplitea:

0: shallow, less than half depth medial condyle;

1: deep.

218. Femur, caudal facies, medial view, internal edge of distal end of shaft:

0: smoothly curving, continuous to condyle, e.g. *Leipoa*;

1: interrupted by caudally prominent crista supracondylaris medialis;

2: crista supracondylaris medialis short or lacking and medial profile notched.

219. Femur, distal end, caudal view, trochlea fibularis, distal extent:

0: equal with condylus lateralis;

1: shorter, merges distally with the lateral side of the condyle proximal to the distal end of the condyle forming a notch.

220. Femur, trochlea fibularis, form and orientation of articular surface:

0: directed caudally, roughly parallel to shaft, with lateral margin proximally merging with lateral facies of shaft smoothly cranial to the impressio ansa m. iliofibularis caudalis;

1: proximal part rotated cranially and facet directed proximally at low angle to shaft, and forms a prominence markedly offset from the lateral facies.

References

Worthy TH, Degrange FJ, Handley WD, Lee MS. 2017. The evolution of giant flightless birds and novel phylogenetic relationships for extinct fowl (Aves, Galloanseres). *Royal Society Open Science* **4**:170975.

Agnolín FL, Brissón Egli F, Chatterjee S, Garcia Marsà JA, Novas FE. 2017. Vegaviidae, a new clade of southern diving birds that survived the K/T boundary. *The Science of Nature* **104**(11‒12):87.
